# Expectation vs. reality: How stereotypes and expectation disconfirmation affect job evaluations in online labor markets

**DOI:** 10.1371/journal.pone.0334630

**Published:** 2025-11-04

**Authors:** Diana Tran Nhat, Timm Teubner

**Affiliations:** 1 Department of Digital Service Engineering, Institute of Technology and Management, Faculty VII, Technische Universität Berlin, Berlin, Germany; 2 Einstein Center Digital Future, Berlin, Germany; University of Amsterdam, NETHERLANDS, KINGDOM OF THE

## Abstract

In online labor markets, reputation determines both job opportunities and pay and even small disparities can translate into significant economic differences. While gender-related bias in reputational metrics such as likes, reviews, and ratings has been empirically documented, the mechanisms through which they arise remain insufficiently understood. This study presents results from an online experiment, varying workers’ gender, domain, and performance, to investigate how expectations about workers are formed and how they affect subsequent evaluations. Drawing on Expectation Disconfirmation Theory and Role Congruity Theory, we test whether the effect of disconfirmation (i.e., discrepancies between expected and actual performance) on evaluations varies with congruity (i.e., stereotypical fit between gender and domain). Contrary to our hypothesis, workers in congruent settings do not elicit higher expectations than those in incongruent roles. Similarly, the effect of positive and negative disconfirmation does not vary with congruity. However, exploratory analyses suggest that congruity does affect expectations and evaluations when individuals hold strong gender-domain associations. In case of stereotypical associations, individuals expect higher performance of workers in congruent domains and evaluate them more leniently if they fail to meet expectations. Our findings contribute to understanding why identical performances might be judged differently depending on gender-domain associations, expectations, and disconfirmation.

## 1. Introduction

Online labor markets (OLMs) have experienced significant growth in recent years, transforming work arrangements and creating new opportunities for millions of individuals worldwide [1]. By offering flexible, on-demand jobs, platform work has become particularly attractive to people who need to balance work with caregiving responsibilities – often women [2]. Compared to jobs in the offline labor market, virtual tasks, such as programming, designing, and translation, are characterized by a higher level of anonymity and involve fewer personal interactions. Theoretically, this should reduce the salience of demographic features and thereby decrease susceptibility to gender bias and discrimination.

However, there is evidence of gender disparities across various dimensions of worker reputation [3]. For instance, men receive more views, likes, and responses than their female counterparts for design work [4]. Moreover, there are differences in both the quantity and quality of ratings women and men receive, with women being rated more positively in some job categories but less favorably in others [5]. Some research shows that while high-performing men and women are evaluated equally, low-performing women are rated more harshly than their male counterparts [6,7]. Overall, previous work provides evidence of gender bias in online reputation, yet there is no clear consensus on when or under what conditions such bias occurs.

While gender is a salient social category, its interpretation is shaped by other contextual cues. Role Congruity Theory [RCT; 8] argues that bias does not simply arise because someone is male or female, but because their gender is perceived as misaligned with the expectations typically associated with the role or domain. Building on this perspective, we do not consider gender in isolation and as a direct source of bias but instead focus on *congruity*, that is, the perceived fit between a worker’s gender and the domain in which they are active. Stereotypes can influence expectations of how men’s and women’s competencies differ for specific domains, especially in those that are (or have been) dominated by a certain gender [9,10]. According to Expectation Disconfirmation Theory [EDT; 11,12], the role of these (biased) expectations in evaluations plays out in a twofold way: First, initial expectations directly impact evaluations, with higher expectations generally leading to higher evaluations (e.g., due to anchoring effects and confirmation bias). Second, expectations serve as a reference point for performance assessments, where disconfirmation of these expectations also affects evaluations.

Although EDT offers a framework for understanding these evaluation processes, it does not theorize about the role of social context, such as the congruity between a worker’s gender and domain, in shaping how these expectation disconfirmations are interpreted. Yet, stereotypes may not only influence the formation of expectations but also how new information is processed. As such, an identical gap between expected and actual performance may be judged differently depending on whether the worker is perceived to “fit” the stereotypical profile of their domain. Importantly, exceeding expectations and falling short of them are not symmetrical phenomena and hence elicit distinct evaluative responses depending on congruity. When a worker is seen as congruent with the domain, exceeding expectations is consistent with the stereotype of a highly competent “congruent” worker and may therefore reinforce existing stereotypical beliefs. In contrast, when a worker is perceived as incongruent, the same positive disconfirmation may be deemed more atypical, thus challenging stereotypical expectations of a less competent “incongruent” worker. This, in turn, may affect how strongly the disconfirmation of expectations impacts evaluations. Despite its theoretical and practical significance, it has not been explored whether the rewards and penalties for over- and underperformance depend on congruity, leaving unexplained why identical worker performances can be judged differently, beyond overt discrimination.

Therefore, further differentiating the effect of expectation disconfirmation is crucial for a deeper understanding of the nuances of congruity-based bias in evaluations. In this paper, we thus investigate how the interplay between individuals’ expectations and workers’ gender, domain, and performance shapes evaluations. Using an online experiment (*n* = 198), we study how congruity affects expectations about workers’ performance and the effect of expectation disconfirmation on evaluations. Overall, we find that congruity does not impact expectations nor moderate the effects of disconfirmation on evaluations. However, our exploratory analyses suggest that congruity-based bias does occur – but only among individuals with strong gender-domain associations. With these findings, we make two key contributions. First, we contribute to RCT by uncovering that the emergence of congruity-based bias depends on the strength and direction of gender-domain associations. Second, by combining RCT and EDT, our study offers a theoretical lens to reconcile mixed evidence on gender-based evaluation bias in the platform economy.

## 2. Related work

The impact of gender in traditional offline labor markets has been researched extensively, shedding light on disparities in wages, promotions, and performance evaluations [e.g., 13–15]. While the causes of these gender gaps are complex, many explanations center around stereotypes that affect both men and women, but in distinct forms [16]. Descriptive stereotypes refer to notions about how men and women typically *are*, whereby male stereotypes are based on agency (e.g., associating men with ambition, assertiveness, and analytic expertise), whereas female stereotypes revolve around communality (e.g., associating women with kindness, collaboration, and intuition) [17]. As a result, this stereotype of men being more ambitious and assertive than women leads to the perception that men are more suitable for leadership positions. Conversely, women are seen as kinder and more collaborative and therefore, more fitting for social work.

In addition, there are prescriptive stereotypes that comprise norms of how men and women *should be* and how they *should behave*. While descriptive and prescriptive stereotypes refer to distinct concepts, there is also an overlap, as characteristics that are perceived to be typically male or female can also simultaneously constitute norms for appropriate behavior [18]. As norms for men and women differ, the same behavior can be judged differently depending on gender. For example, a female leader acting agentically may be perceived less positively than a male leader exhibiting the same behavior [19].

With the rise of OLMs, scholars have been increasingly interested in whether gender-related issues are also prevalent in the digital space. Indeed, there is a stream of research indicating the presence of a gender pay gap on platforms such as Uber [20,21], Upwork [22], and Freelancer [23]. Moreover, job segregation is also reflected in the online world, with male workers being overrepresented in stereotypically male fields (e.g., programming, engineering) while female workers predominate in traditionally female jobs (e.g., writing, customer services [22]). Naturally, some of these issues might be due to worker self-selection (e.g., choosing stereotypical job categories, acquiring different skills) and algorithmic bias (e.g., in recommendation systems, search results), which is difficult to disentangle from stereotype-based discrimination only based on observational data. In this paper, we will thus leverage an experimental approach to shed light on the causal effects of congruity perceptions within the decision-making process of clients.

One of the first decisions for clients is who to hire for their project, an important choice that could be prone to bias. On a non-disclosed OLM, Chan and Wang [24] observed that female workers are generally preferably hired as women are perceived as more attractive, cooperative, and trustworthy than men. In contrast, Galperin [25] found no general pro-male or pro-female bias on Nubelo (the platform was acquired by Freelancer in 2016). Instead, he observed that women have lower hiring chances for male-typed jobs that are associated with technical skills but are, to a similar extent, preferred for female-typed jobs. Leung and Koppman [26] investigated the decision *not to hire* on Elance (the platform merged with oDesk in 2014 and was renamed to Upwork in 2016). On this platform, clients post jobs that workers apply to, forming an applicant pool. The authors found that with an increasing share of applicants whose gender does not match the job stereotype, clients are more likely to not hire *anyone* as they believe that the pool does not include suitable job candidates.

While hiring choices are highly important as they determine whether and with whom a transaction is initiated, the performance evaluation at the end of a transaction also entails significant consequences for workers. On platforms that are characterized by a high level of anonymity, ratings support customers in their decisions regarding whom to trust, incentivize trustworthy behavior, and punish low quality and dishonesty [27]. As ratings provide credible information about workers’ skills, competencies, experience, and trustworthiness, clients are found to rely heavily on these in their hiring decisions [28–30]. In fact, reputation impacts consumer behavior more strongly than the perceived trustworthiness of users’ profile pictures or the stake of a transaction [31].

Therefore, reputation and hiring decisions are closely linked in a positive feedback loop whereby a good reputation increases workers’ hiring chances, which, in turn, will further their reputation. Additionally, ratings affect workers’ *ranking* [32], determining their likelihood of being found by clients in the first place [33]. Beyond their role in attracting demand, ratings also exert a positive effect on workers’ wages [34]. Due to the immense significance of worker reputation, a growing body of work has investigated whether online ratings exhibit gender bias.

For example, Wachs et al. [4] found that men present more work on the online design community Dribbble, for which they receive more views, likes, and responses than women. They explain that listing “male” skills and creating “male” images partially contribute to this success, irrespective of gender, suggesting that women may self-select in acquiring different skills and producing different designs. Moreover, while the aforementioned studies on hiring chances demonstrated a reward for gender-stereotype match [24–26], the reverse effect was found for online reputation, at least in certain cases. For instance, Hannák et al. [5] observed that women receive more reviews and substantially higher rating scores in several male-dominated job categories on Fiverr (e.g., Databases, Web Analytics, and Financial Consulting). Yet, their results remain inconclusive as in all other job categories, opposing effects were found. On TaskRabbit, a platform that intermediates predominantly manual work, women receive fewer reviews than men with similar work experience [5].

Another line of research investigated whether the emergence of bias is contingent on the performance of workers. In an experimental study, Jahanbakhsh et al. [7] found that high-performing men and women were evaluated equally. However, when work performance was low, women were rated more unfavorably than men. Greenwood et al. [6] delivered corroborating evidence from an experiment in which female drivers of a fictitious ridesharing app were disproportionately penalized only when quality was low. Conversely, when quality was high, gender did not have an impact on ratings. In contrast, Thebault-Spieker et al. [35] also varied the performance of workers in a series of four experiments but did not find gender bias in any setting. Their analyses suggest that if male and female workers were rated differently, the effect would be lower than 0.2 stars.

Beyond static conceptions of stereotyping and bias, other work has explored how gender discrimination evolves over time and with accumulating information. Bohren et al. [36] developed a theoretical framework and conducted a field experiment in an online mathematics Q&A forum, showing that women initially face discrimination in evaluations. However, this bias reverses once performance histories accumulate. Over time, women are favored over men with equivalent evaluation records, a pattern that is interpreted as evidence of biased beliefs rather than preference-based discrimination. These findings inform our own investigation by highlighting the critical role of expectations in shaping evaluations.

**Fig 1:**
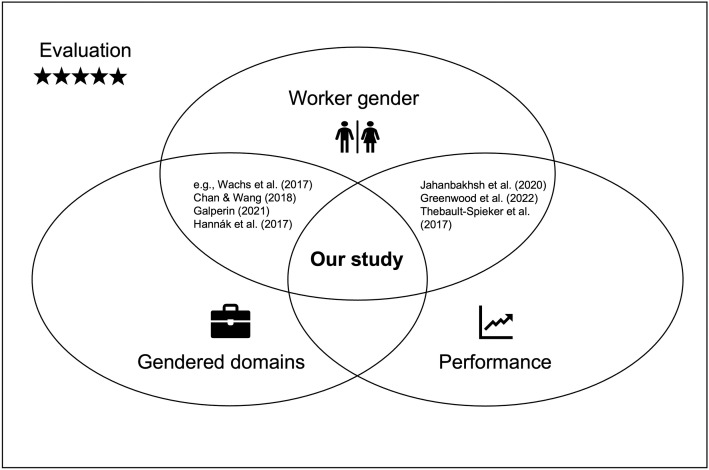
Mapping of existing literature and our study.

To conclude, prior work on gender bias in workers’ reputations comes to somewhat conflicting results but points out important moderating factors. Specifically, it seems that bias is evoked when workers’ gender does not match the stereotypes associated with a domain or job. Further, bias can be amplified or mitigated, depending on the level of performance. However, none of the aforementioned studies examined the joint influence of both job domain *and* performance on evaluations (see Fig 1). In the next chapter, we will build upon these findings by considering both factors to derive our hypotheses.

## 3. Hypothesis development

In the following, we seek to reconcile the results of prior studies by considering the moderating role of worker performance and gendered expectations. To do so, we draw on EDT [11,12] and RCT [8].

Oliver [11] developed EDT to assess the evaluation of product performance through ratings. In a nutshell, EDT posits that initial expectations about a product or service, as well as the confirmation or disconfirmation of these expectations, affect client satisfaction. The theory’s first assertion is that higher expectations will result in higher levels of satisfaction, an effect that is (relatively) independent of service quality and performance. In addition, expectations serve as a benchmark for actual performance, which can result in confirmation of expectations, when performance exactly matches expectations, in negative disconfirmation of expectations, when performance falls short of expectations, or in positive disconfirmation of expectations, when performance exceeds expectations [37]. In the following, *disconfirmation of expectations* is referred to as *disconfirmation*. Likewise, *confirmation of expectations* is referred to as *confirmation*. As expectations and the disconfirmation thereof are key to EDT, it is crucial to understand how expectations are formed, particularly in light of gender stereotypes.

To theorize on gender-related effects on expectation, we draw on RCT by Eagly and Karau [8], which originally analyzed prejudice toward female leaders. Central to RCT is the notion of *congruity*, that is, the perceived fit between a person’s gender and their role in a given context. Importantly, RCT posits that bias does not emerge from gender per se – but due to perceived incongruity between gender and role. Therefore, we will focus on the (mis-) alignment of worker gender and the stereotypes about a domain. Incongruity, that is, the perceived mismatch between workers’ gender and their domains, leads to two forms of prejudice according to RCT [8]. First, workers perceived as incongruent are seen as less suitable for the role in question. Second, incongruent workers engaging in counter-stereotypical behavior are evaluated less positively than congruent workers.

To derive our hypotheses, we synthesize EDT and RCT. A natural intersection of both theories occurs at the elements of expectation and evaluation. Based on that, we distinguish between the expectation formation phase and the evaluation phase. In the first stage, clients form expectations about workers’ competence and trustworthiness to decide whom to hire. On OLMs, eye-catching profile pictures are highly salient and visible, prompting gender categorization and judgments of congruity, often without conscious awareness [38,39]. In fact, bias can operate subconsciously despite efforts to avoid overt discrimination [40]. Although profile information provides as powerful indicators of workers’ competence, such as ratings, reviews, and employment history, these are noisy signals, allowing room for interpretation. As gender is a cultural frame that shapes expectations and perceptions, information can be interpreted differently depending on congruity [41].

Specifically, due to confirmation bias, clients are likely to assimilate information to corroborate their initial impression of workers [42]. For one, selective attention to stereotype-consistent information may reinforce perceptions of congruity. In addition, stereotype-inconsistent information could be assigned less weight in the expectation formation process. When facing ambiguity, individuals also tend to fill gaps in information with assumptions that are in line with their beliefs [43,44]. Therefore, we hypothesize that despite the abundance of information on profiles, clients will have higher expectations regarding the performance of workers in stereotypically congruent domains. As expectations also affect hiring decisions, our hypothesized effect would also explain the hiring bias in gendered domains observed by Galperin [25], Chan and Wang [24], and Leung and Koppmann [26].

**H**_**1**_: *Stereotypical role congruity increases clients’ expectations about worker performance.*

According to EDT, these (biased) expectations have a positive direct effect on evaluations (Premise 1; P_1_ – “anchoring effect”), while also indirectly affecting them through positive/negative disconfirmation [12]. Specifically, exceeding (falling short of) expectations increases (decreases) satisfaction (Premise 2; P_2a/b_). Hence, we posit that congruity indirectly affects evaluations through these channels. Note that these effects might (at least partially) cancel each other out: While high expectations provide an anchor for high(er) evaluations, they will – ceteris paribus – also yield negative disconfirmation. A way to differentiate these partial effects is hence to account for prior expectations and posterior disconfirmation separately.

In the context of our study, we propose that stereotypical congruity could moderate the effect of disconfirmation on evaluation. Positive and negative disconfirmation are fundamentally distinct and could be contextualized differently, depending on worker-domain congruity. We posit that in some scenarios, disconfirmation may further corroborate biased beliefs, while in others, disconfirmation may refute stereotypical expectations.

**Fig 2:**
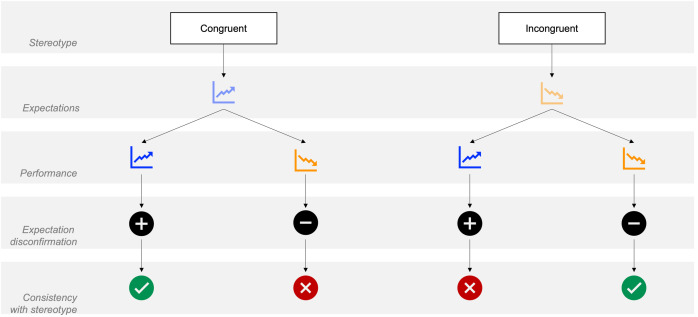
Classification of scenarios based on congruity, expectation, and performance.

On the one hand, workers’ performance can be in stark contrast to clients’ expectations. A contrast can occur when expectations about workers in incongruent roles are low but actual performance is high (i.e., positive disconfirmation). Similarly, workers with domain congruity might fail to meet high expectations (i.e., negative disconfirmation). In both scenarios, the disconfirmation does not align with stereotypes as expectations and actual performance diverge (i.e., low expectations and *high* performance; high expectations and *low* performance; see scenarios marked in red in Fig 2).

Individuals tend to use automatic, heuristic processing unless required or motivated to engage in deliberate, systematic analysis [45]. Performance that clearly contradicts expectations can induce cognitive dissonance that individuals may want to resolve through systematic reasoning [46]. For instance, they may attribute the exceptional performance of workers in incongruent domains to their skills, dedication, but also luck [47]. Conversely, poor performance by workers in congruent roles may be ascribed to external factors such as task complexity, suggesting that individual performance is influenced by various (external) factors beyond gender stereotypes. This process may individuate the worker as clients reconsider their initial stereotypical categorization [42]. Taken together, these deliberate reasoning processes could contribute to a debiasing effect on evaluations.

On the other hand, workers’ performance can also be consistent with clients’ (biased) expectations, most obviously when performance matches expectations. However, disconfirmation may still be in line with stereotypes in certain cases. If workers in congruent domains exceed (already) high expectations, this overperformance (i.e., high expectations and *higher* performance) reinforces stereotypical beliefs (see scenarios marked in green in Fig 2). Likewise, workers in incongruent roles who fail to meet (already) low expectations are consistent with stereotypes. A “disconfirmation” into an already suggested direction would, in this sense, represent an affirmation rather than a challenge of one’s beliefs.

When performance aligns with prior (stereotypical) expectations, it is less likely that individuals shift from their default, that is, an intuitive, automatic mode of processing to a more deliberate and effortful mode [45]. Since the initial categorization of the worker as congruent or incongruent seemingly provided reasonable predictive power regarding their performance, clients are less inclined to rethink their stereotyping of the worker [42]. Furthermore, selective attention to information consistent with initial expectations can result in confirmation bias that may corroborate stereotypes [48]. In addition, consistent information is processed more easily and requires less cognitive effort than inconsistent information [49], increasing susceptibility to bias [50].

Hence, the appreciation of workers’ overperformance might be stronger for those in congruent domains than for their counterparts in incongruent roles. In contrast, positive disconfirmation of workers in incongruent settings is unlikely to yield such disproportionally high rewards. Since their performance is inconsistent with stereotypes, the negative bias of incongruity could be mitigated – but unlikely becomes positive. Thus, we hypothesize that

**H**_**2a**_: *Positive disconfirmation is rewarded more strongly for workers in stereotypically congruent domains than it is rewarded for those in incongruent roles.*

In the same vein, underperforming workers in incongruent constellations will be penalized more harshly than those in congruent settings. Since their underperformance is consistent with stereotypes, clients’ notion of incongruity could be reinforced, further distorting evaluations negatively. Accordingly, we hypothesize that

**H**_**2b**_: *Negative disconfirmation is penalized more strongly for workers in incongruent domains than for those in congruent roles*.

**Fig 3:**
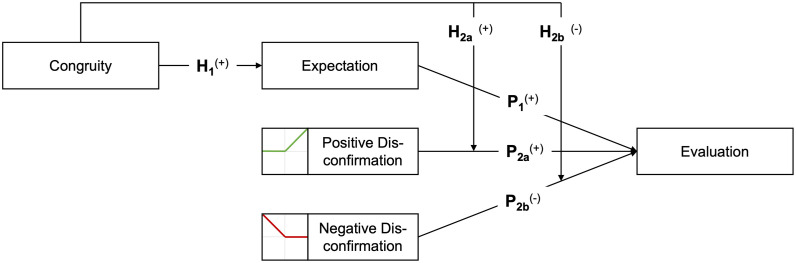
Research model and hypotheses.

The hypothesized effect would be consistent with findings of Greenwood et al. [6] and Jahanbakhsh et al. [7] who only found bias in ratings for low – but not for high performance. Fig 3 summarizes our research model and all hypotheses.

## 4. Method

To evaluate our research model, we conducted a scenario-based online experiment.

### 4.1. Sample

The study was reviewed and approved by the Ethics Committee of the Faculty of Economics and Management (VII), Technical University Berlin. We recruited 201 participants via *Profilic.co* [51] from May 3 to May 4, 2023. Informed consent was obtained electronically from all participants through an active opt-in procedure. Participants indicated their consent by actively clicking “yes” before proceeding to the experiment. Three participants were excluded due to failing the attention checks, resulting in a final sample size of 198. On average, participants took 15.83 minutes to complete the experiment (*median*  = 11.96; *SD  *= 14.33) and earned GBP 3.22. Participants’ age ranged from 20 to 76 years, with a mean age of 27 years (*median  *= 25; *SD* = 7.48). As per our sampling request to the platform, half of all participants were female (50.5%). Most participants identified as white (68%), followed by black (18%), mixed (8%), and Asian (3%).

### 4.2. Treatment design

We employed a full-factorial between-subjects design, in which each participant was exposed to a single treatment condition that varied along three dimensions: the worker’s *gender* (male or female), the *domain* (male- or female-dominated), and the worker’s *performance*. First, the **worker’s gender** was signaled by first names, randomly drawn from the set of Nosek et al. [52], Ben, Paul, Daniel, John, and Jeffrey as male names; and Rebecca, Michelle, Emily, Julia, and Anna as female names. In addition, the worker’s gender was signaled by the AI-generated profile pictures, sourced from *ThisPersonDoesNotExist.com* (see supporting information, S1 Fig and S2 Fig). The depicted individuals’ characteristics (e.g., age, attractiveness, competence) were evaluated by human raters recruited via Prolific.co [51] and by using a Microsoft API (see supporting information, S1 Table).

Second, for the **domain**, we used settings of a car parts shop (stereotypically male-dominated) vs. a clothing store (stereotypically female-dominated). Third, we varied the (textual) description of the worker’s **performance** in the three categories 1) punctuality, 2) layout of the online shop, and 3) product descriptions. In each category, the performance was indicated to be of low, medium, or high quality.

Following Heilman and Chen [53], we used the exact same tasks across all treatments (i.e., designing a shop layout and writing product descriptions). In the same vein, to avoid additional noise, all workers were based in Berlin (Germany), had an hourly rate of EUR 50, and offered the same services (i.e., logo design, web layout design, brand identity design, content for newsletter, blog entries, and product descriptions).

While gender and domain were assigned at random, overall performance was balanced as a uniform distribution. As each of the three performance dimensions (i.e., punctuality, online shop layout, and product description) could take on three quality levels (i.e., low, medium, and high quality), we further classified the overall performance into seven levels (see Table 1). Here, a performance of seven represents the highest appraisal of the worker (high, but sub-perfect quality in all three dimensions; see supporting information, S2 Table) and one the lowest (low quality in all three dimensions). In each row of Table 1, column 2 shows the combinations of dimension-specific evaluations that correspond to each performance level. For example, a performance level of six includes high evaluations in two dimensions and a medium evaluation in one dimension. This could be the case when the worker delivered results on time (high punctuality) and the quality of the online shop layout was high, yet the product descriptions were of medium quality.

**Table 1:** Performance treatments.

Performance	Combinations of evaluations		
7	High quality in 3 dimensions		
6	High quality in 2 dimensions	Medium quality in 1 dimension	
5	High quality in 2 dimensions		Low quality in 1 dimension
	High quality in 1 dimension	Medium quality in 2 dimensions	
4		Medium quality in 3 dimensions	
	High quality in 1 dimension	Medium quality in 1 dimension	Low quality in 1 dimension
3		Medium quality in 2 dimensions	Low quality in 1 dimension
	High quality in 1 dimension		Low quality in 2 dimensions
2		Medium quality in 1 dimension	Low quality in 2 dimensions
1			Low quality in 3 dimensions

After randomly drawing a performance level, one variant of the performance description was selected. Table 2 presents the number of observations in each experimental condition.

**Table 2:** Number of observations per experimental condition.

Worker	Domain	Stereotype	Performance	# of observations	
Male	Car	Congruent	1	55	8
			2		9
			3		6
			4		4
			5		9
			6		7
			7		12
	Fashion	Incongruent	1	51	10
			2		8
			3		10
			4		7
			5		6
			6		6
			7		4
Female	Car	Incongruent	1	49	9
			2		5
			3		6
			4		9
			5		8
			6		7
			7		5
	Fashion	Congruent	1	43	3
			2		4
			3		6
			4		11
			5		5
			6		10
			7		4

### 4.3. Task and stimulus material

Participants were instructed to assume the role of an online shop manager who has been appointed to establish an online shop. For this purpose, the manager has hired a worker to design the layout of an online shop and to write product descriptions. Should the online shop meet all requirements and be launched on time, the manager would receive a bonus payment of EUR 1,500. This bonus would be reduced as the shop deviates from the agreed specification, and the more the launch is delayed. As the successful launch of the shop relied on the worker’s results, their performance directly affected the manager’s bonus payment.

**Fig 4:**
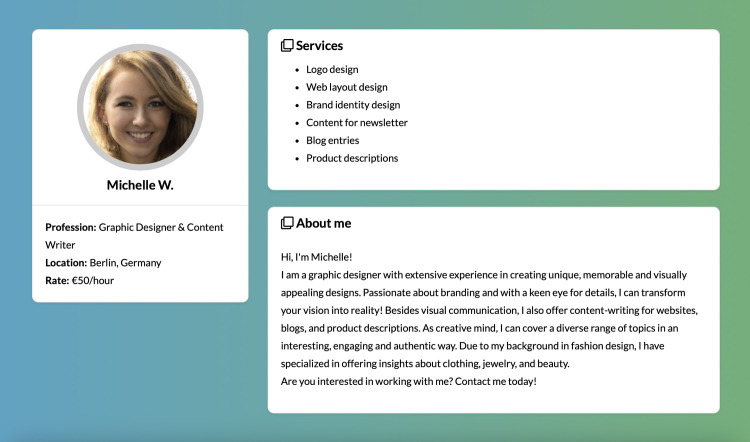
Exemplary profile of freelancer.

Subsequently, the participants were presented with the profile of the worker, which was stylized and informed by the design of freelancing platforms (see Fig 4). The profile included an AI-generated image of the fictitious worker, sourced from *ThisPersonDoesNotExist.com*. After having seen the profile, participants indicated their expectations of the worker’s performance. Next, participants read an e-mail from a team member that described the actual performance, particularly regarding the worker’s timeliness and the quality of the shop layout and product descriptions.

The e-mail summarized which aspects needed improvement and concluded with an estimate of the delay in the shop launch (see supporting information, S2 Table). Participants were then informed of the reduction of their bonus. Note that even the highest performance level was not described as perfect and some potential for improvements was highlighted. As a result, even in the best-performing condition, participants received a small bonus reduction.

Finally, the participants were asked to rate the worker on a 5-star rating scale. In addition, we included an incentivized task in which the participants estimated the average rating of the other participants. Note that a common issue with subjective evaluations is that there is no feasible way to prevent random responses as there is no incentive to state one’s actual evaluation truthfully (but conversely, there is also no incentive to lie). Hence, we offered an incentive for the prediction to reduce hypothetical bias and increase participants’ engagement. Moreover, it enabled us to identify any potential systematic differences compared to non-incentivized behavior. After finishing the main part of the experiment, participants filled out a survey which also included realism, attention, and manipulation checks.

### 4.4. Incentives and payouts

Participants received a fixed payment of GBP 0.50. In addition, to increase participants’ emotional engagement, we paid out a share of the (hypothetical) bonus that the manager received in the scenario. As the bonus depended on the worker’s results, their performance had a direct impact on participants’ utility. To strengthen the value of the bonus, we displayed it in an actual currency (EUR) [54]. This bonus was then paid out with a conversion rate of GBP 0.02/ EUR 100. For the highest appraisal levels (work of high, yet sub-perfect quality; Performance 7), the bonus was reduced from the initially promised EUR 1,500 to EUR 1,400. For each decrease in performance, the bonus incrementally decreased in steps of EUR 200 until the minimum of EUR 200 (Performance 1). Hence, the share paid out to the participants ranged from GBP 0.04 to GBP 0.28.

In the incentivized task, participants could receive a performance-based bonus for estimating the average evaluation of other participants. If the participants matched the average evaluation exactly, they received a bonus of GBP 0.60. For each decimal that their estimate deviated from the correct answer, their bonus was reduced by GBP 0.10 (to a minimum of zero).

### 4.5. Measurements

Our main variable of interest is how participants **evaluated** the worker’s performance on a 5-star rating scale in steps of 0.1 (i.e., 1.0, 1.1, 1.2, …, 4.9, 5.0). Using the same scale, we asked participants to provide their best estimation of the average evaluation of all other participants.

Moreover, we measured participants’ **expectations** about the worker’s performance on a 7-point Likert scale, which we chose to match the seven performance levels, facilitating the comparison between expected and actual performance. Specifically, we used a four-item construct to capture participants’ anticipations concerning the worker’s punctuality, fulfillment of demands, layout quality, and product descriptions. Although performance was operationalized across three dimensions, we included the fourth item (“fulfillment of demands”) to measure participants’ overall expectation of the worker’s success. While this item does not directly correspond to a specific performance dimension, it was intended to reflect a more holistic assessment of participants’ expectations. We verified the construct’s reliability (Cronbach’s alpha = .78; threshold based on Nunally and Bernstein [55]) before we averaged participants’ responses across the four items into a single expectation score for subsequent analysis.

Additionally, to infer **disconfirmation** of expectations, we distinguish between positive (performance > expectation) and negative (performance < expectation) disconfirmation. To allow for different effects/slopes in the regression analysis, we created two variables for positive and negative disconfirmation. If the difference (Δ = performance – expectation) was greater than zero, the variable capturing positive disconfirmation took on the respective values of the difference while it was zero, otherwise. Negative disconfirmation was calculated accordingly.

As an additional measure, participants conducted the ***Implicit Association Test* (IAT)**, a timed sorting task to measure gender-domain associations. We selected the IAT due to its higher predictive validity compared to self-report measures, which are susceptible to socially desirable answers, particularly in contexts involving sensitive topics such as gender bias [56]. Initially, we intended to use the IAT scores to infer participants’ subjective perception of congruity in the presented scenario. However, due to the IAT’s low test-retest reliability, we ultimately relied on a definition of stereotypical **congruity** based on the exogenous treatment variables, that is, worker gender (male/female) and domain (cars/fashion). Drawing on conventional stereotypes, we assumed female (male) workers in the fashion (car) domain to be perceived as congruent and likewise female (male) workers in the car (fashion) domain to be seen as incongruent (see Table 3). This dual definition of congruity is important for our analyses, as the interaction term between the binary variables gender and domain only captures one of the two congruent combinations (i.e., when both variables are 1).

**Table 3:** Construction of the variable *Congruity.*

	Domain car	Domain fashion
**Worker male**	Congruent	Incongruent
**Worker female**	Incongruent	Congruent

Beyond that, we surveyed participants about their decision-making in the experiment and how realistic they deemed the scenario in the experiment. We also asked how they felt about the reduced bonus due to the worker’s performance. Furthermore, the survey included questions about participants’ online rating behavior and demographic information.

### 4.6. Randomization check

To test whether the assignment of the treatment variables worker *gender*, *domain*, and *performance* was indeed random, we searched for potential disparities between participants across treatment groups. Specifically, we ran a set of ordinary least squares (OLS) regressions with the participants’ age, perceived realism of the scenario, and online rating frequency as dependent variables. Moreover, we used the IAT test’s standardized *d*-scores (following Greenwood et al. [57]), which measure the relative strength of stereotypical (e.g., male-cars) versus counter-stereotypical (e.g., female-cars) associations.

The independent variables comprised the main treatment variables *worker gender* (binary, male = 0, female = 1), *domain* (binary, stereotypically male = 0, stereotypically female = 1), and *performance* (level 1–7). We also tested all resulting 2 × 2 and 2 × 2 × 7 interactions. As summarized in Table 4, no statistically significant differences between participants across the treatment groups were detected. In addition, we also ruled out any significant correlation between performance (exogenous treatment manipulation) and participants’ expectation (*p *= .239).

**Table 4:** OLS regression results for randomization check.

Variable	Participant age	IAT *d*-score	Perceived realism	Rating frequency
Worker gender ^1)^	−1.833 (3.157)	−0.159 (0.141)	−0.031 (0.401)	−0.804 (0.641)
Domain ^1)^	1.626 (3.005)	0.117 (0.135)	−0.063 (0.385)	−0.374 (0.615)
Performance	−0.345 (0.450)	−0.028 (0.020)	−0.058 (0.058)	−0.129 (0.092)
Worker gender ^1)^ × Domain ^1)^	−6.066 (4.849)	−0.130 (0.218)	0.027 (0.618)	0.729 (0.988)
Worker gender ^1)^ × Performance	0.448 (0.698)	0.037 (0.031)	−0.004 (0.089)	0.181 (0.142)
Domain ^1)^ × Performance	−0.045 (0.697)	−0.021 (0.031)	−0.030 (0.089)	0.088 (0.143)
Worker gender ^1)^ × Domain ^1)^ × Performance	2.012 (1.088)	0.049 (0.049)	0.096 (0.139)	−0.231 (0.223)
Constant	27.140**** (2.128)	0.397*** (0.096)	5.860*** (0.273)	4.379*** (0.436)
**Observations**	195 ^2)^	198	198	198
**Adjusted *R*** ^**2**^	0.069	0.022	−0.003	−0.014
**Res. std. error**	7.219 (*df *= 187)	0.326 (*df* = 190)	0.927 (*df* = 190)	1.481 (*df* = 190)
***F*-statistic**	3.061 (*df* = 7; 187)	1.641 (*df* = 7; 190)	0.916 (*df* = 7; 190)	0.599 (*df* = 7; 190)

**Note:** Standard errors in parentheses; *p < .05; **p < .01; ***p < .001.

1)0 = (stereotypically) male, 1 = (stereotypically) female.

2)For three participants, information on age was not available.

## 5. Results

### 5.1. Overview

**Fig 5:**
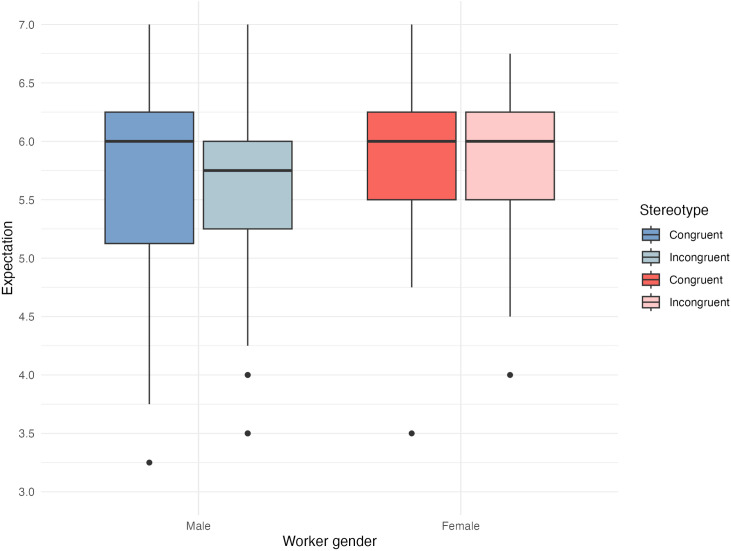
Boxplot of expectation separated by worker gender and congruity.

First, we consider participants’ expectations about workers’ performance. Overall, expectations ranged between 3.25 and 7.00 (on the 4-items 1–7 Likert scale; mean of 5.70; *SD* = 0.78). Differentiating by congruity, we see that neither male nor female workers in stereotypically congruent constellations evoke higher expectations than in incongruent constellations (see Fig 5). On average, expectations in congruent and incongruent constellations were almost identical (5.78 vs. 5.63 on average; *t*(196) = 1.39, *p* = .167).

Subsequently, we take another perspective and instead of focusing on congruity, we estimate a set of baseline regressions to examine the direct effect of worker gender, domain, and their interaction on expectations, holding workers’ and participants’ characteristics constant (see S4 Table in the supporting information for full results). Across all models, neither gender, domain, nor their interaction significantly predicts expectations.

**Fig 6:**
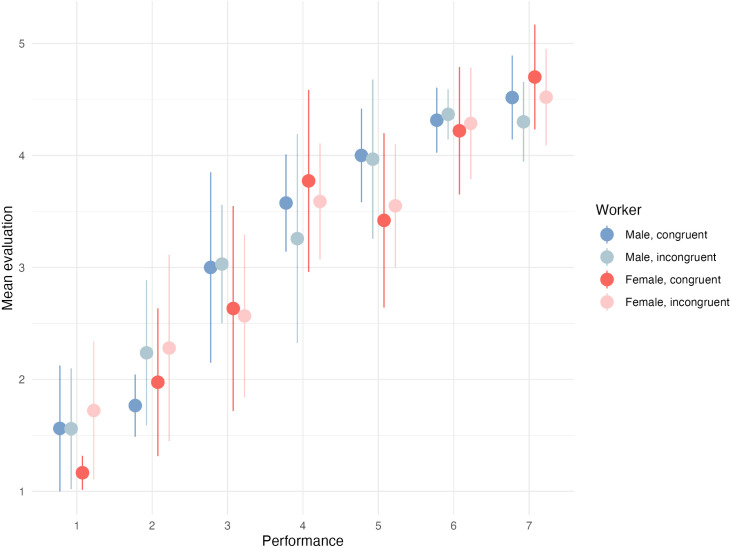
Mean evaluation and standard deviation by performance and congruity.

We then went on to analyze how the participants rated workers. Fig 6 shows that across both treatment groups, evaluations increase with performance (almost linearly). However, we do not see any evidence for differences in evaluations regarding congruity, neither for male nor for female workers.

In addition, we investigate the direct effects of worker gender, domain, and their interaction on evaluations while controlling for various worker and participant features (see S5 Table in the supporting information for full results). The results indicate that neither the workers’ gender, domain, nor the interaction between these two variables significantly influenced ratings.

As a robustness check, we asked participants to “guess” how *other participants* would evaluate an identical worker in the same situation and incentivized this guess with an additional bonus payment, which depended on how close their guess would fall to other participants’ actual evaluations. Importantly, participants’ own assessments and their guesses about other participants’ assessments were highly correlated (*r *= .906; *p* < .001), suggesting that participants’ own evaluations can be considered robust.

**Fig 7:**
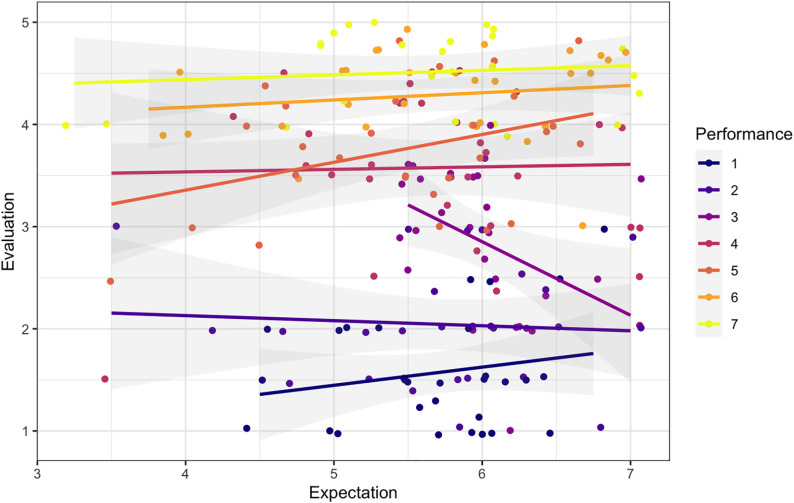
Effect of expectations on evaluation by performance.

Moreover, we examined how expectations affect evaluation while controlling for performance. As shown in Fig 7, while performance has a strong effect, expectations appear to have no systematic effect on evaluations across performance levels (β = −.050, *p* = .644). This stands in contrast to EDT but may be explained by our experimental setup. Participants only held their expectations briefly, as they learned about the worker’s actual performance almost instantly after they indicated their expectations. This ephemerality, of course, leaves little room for expectations to “sink in” and thus to exert a more lasting effect. In contrast, clients in real-world settings usually hold expectations over a longer period (e.g., hours, days, or even weeks), which may increase the salience of those expectations and their resulting anchoring effect.

### 5.2. Hypothesis testing

Following up this first visual assessment, we now estimate a set of OLS regression models to test our hypotheses (Table 5). In Model (i), we analyze how stereotypical worker-domain congruity affects expectations (H1). The results indicate no significant relationship (β = .143, *p* = .189; H1 not supported). A variation of Model (i), including the effect of gender, domain, and gender-domain interaction also shows no significant effects (see supporting information, S4 Table).

**Table 5:** OLS regression results for Hypothesis 1 and 2.

	Expectation	Evaluation	
	(i)	(iia)	(iib)
Congruity	.143 (.108)	−.015 (.087)	.033 (.163)
Performance		.539*** (.068)	.538*** (.068)
Positive disconfirmation		−.288** (.083)	−.333** (.118)
Negative disconfirmation		.033 (.070)	−.018 (.074)
Positive disconfirmation ^1)^ × Congruity			.081 (.156)
Negative disconfirmation ^2)^ × Congruity			−.035 (.055)
Worker female	.171 (.108)	−.019 (.087)	−.015 (.087)
Participant female	−.436*** (.108)	.152^+^ (.090)	.140 (.090)
Gender match ^3)^	−.003 (.108)	.036 (.086)	.029 (.087)
Constant	5.765*** (.118)	1.153** (.407)	1.144** (.411)
**Observations** ^4)^	195	195	195
**Adjusted *R*** ^**2**^	.079	.749	.748
**Res. std. error**	.753 (*df *= 190)	.597 (*df *= 187)	.598 (*df* = 185)
***F*-statistic**	5.142 *** (*df *= 4; 190)	83.737 *** (*df *= 7; 187)	65.043 *** (*df* = 9; 185)

**Note:** Standard errors in parentheses; + *p* < .1; *p < .05; **p < .01; ***p < .001

1)Underestimation of performance

2)Overestimation of performance

3)Binary variable indicating whether participant and worker were of the same gender.

4)For three observations, information on gender was not available.

Our visual inspection suggested that expectation has no significant effect on evaluations (see Fig 7), which we confirmed statistically using an OLS regression model (β = −.050, *p* = .644; Premise 1 not supported). Although our research model, grounded in EDT, centers around the role of expectations and disconfirmation in shaping evaluations, these findings led us to revise our modeling approach. Since disconfirmation is derived from the difference between expectation and performance, including all three variables in the same model would result in an overdetermined model. In Models (iia) and (iib), we hence used performance (as well as disconfirmation) as predictors instead of expectations directly. Nevertheless, the influence of expectation still indirectly remains through the disconfirmation variable, which incorporates expectation by design.

Model (iib) tests our premises as well as H2a and H2b. Note that we now consider disconfirmation while controlling for performance (instead of expectations). Therefore, for a given performance level, the disconfirmation variable refers to the under- and overestimation of performance. Positive disconfirmation, in this sense, must be interpreted as *underestimation* while negative disconfirmation implies *overestimation*. The effect of positive disconfirmation (i.e., underestimation) is significant and negative (β = −.333, *p* = .005). Thus, when (ex-ante) expectations fell below (a fixed level of) performance, these lower expectations act as an anchor, reducing evaluations. However, this effect does not significantly vary with congruity (β = .081, *p* = .604; H2a not supported). Negative disconfirmation (i.e., overestimation) does not affect evaluations (β = −.018, *p* = .811), again with no significant interaction with congruity (β = −.035, *p* = .520; H2b not supported).

**Fig 8:**
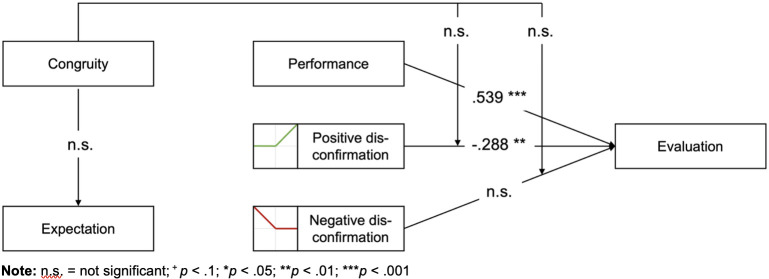
Evaluation of the research model.

To conclude, congruity neither influences expectations nor moderates the effects of positive or negative disconfirmation. Fig 8 summarizes these findings. Note that as the interaction between congruity and disconfirmation is not significant, the figure displays the coefficients of Model (iia), which excludes the interaction terms.

### 5.3. Alternative specifications of the models

Given the possibility that our exogenous manipulation of congruity might not have been perceived as such by participants, we now explore whether individual differences in gender-domain associations might provide additional predictive power. While our primary analysis assumed that all participants would view male-car and female-fashion pairings as congruent, this assumption might be overly simple. Individuals may differ in the strength and/or direction of their gender-domain associations, which could influence whether a given worker-domain constellation is perceived as congruent or not. For instance, it is conceivable that some participants would view female workers in the car domain as congruent.

To examine this possibility, we analyzed participants’ reaction times in the IAT using the *d*-scores (see Section 4.6). Most participants (i.e., 85%) exhibited faster response times for stereotypical worker-domain constellations (i.e., male workers in the car domain/female workers in the fashion domain). However, a non-negligible share of participants also exhibited faster responses for non-stereotypical constellations.

Note that this supplementary analysis is exploratory and should be interpreted with caution. Although the IAT is widely used to measure implicit gender attitudes [e.g., 58–60], its test-retest reliability at the individual level has been reportedly limited [61]. Nonetheless, given that congruity is inherently a perceptual construct, we believe it is meaningful to consider individual variation in how congruity might be *perceived*. To account for these individual differences, we tested whether the effect of congruity on expectations and evaluations depends on each participant’s individual *d*-score. Thus, we treat congruity as the experimental manipulation and the *d*-score as a continuous moderator, capturing the direction and strength of participants’ associations between gender and domain. The results of this analysis are summarized in Table 6.

**Table 6:** OLS regression results, including the interaction between congruity and *d*-score.

	(iii)	(iv)
	Expectation	Evaluation
Congruity	−.073 (.152)	.278 (.233)
*d*-score	−.093 (.229)	−.218 (.343)
Performance		.547*** (.069)
Positive disconfirmation ^1)^		−.352** (.120)
Negative disconfirmation ^2)^		−.005 (.083)
Congruity × *d*-score	.663* (.330)	−.652 (.512)
Positive disconfirmation ^1)^ × Congruity		−.096 (.199)
Negative disconfirmation ^2)^ × Congruity		−.179* (.085)
Positive disconfirmation ^1)^ × *d*-score		−.076 (.238)
Negative disconfirmation ^2)^ × *d*-score		−.013 (.114)
Positive disconfirmation ^1)^ × Congruity × *d*-score		.547 (.436)
Negative disconfirmation ^2)^ × Congruity × *d*-score		.377* (.178)
Worker female	.140 (.108)	.003 (.088)
Participant female	−.430*** (.107)	.149 (.091)
Gender match ^3)^	.002 (.107)	.021 (.088)
Constant	5.805*** (.142)	1.162** (.434)
**Observations** ^4)^	195	195
**Adjusted *R*** ^**2**^	.098	.753
**Res. std. error**	.745 (*df *= 188)	.592 (*df *= 179)
***F*-statistic**	4.497 *** (*df* = 6; 188)	40.450 *** (*df *= 15; 179)

**Note:** Standard errors in parentheses; + *p* < .1; *p < .05; **p < .01; ***p < .001

1)Underestimation of performance

2)Overestimation of performance

3)Binary variable indicating whether participant and worker were of the same gender.

4)For three observations, information on gender was not available.

Model (iii) reveals a significant interaction between congruity and participants’ IAT *d*-score (β = .663, *p* = .046), suggesting that the effect of congruity on expectations depends on the strength and direction of a participant’s gender-domain associations. The main effect of congruity, capturing the effect when the *d*-score is exactly zero (i.e., when the reaction time to stereotypical gender-domain combinations is exactly as fast as that to counter-stereotypical word pairings), is not significant (β = −.073, *p* = .629). This result is theoretically coherent: when individuals do not associate either gender more strongly with a given domain, the manipulation of congruity should have little influence on their expectations.

While exploratory, these findings are consistent with the underlying rationale of H1, that the *perceived* congruity affects expectations. In particular, results suggest that when participants’ implicit associations are stereotypical (*d* > 0), then workers in a male-cars or female-fashion scenario evoke higher expectations than their counterparts in female-cars or male-fashion constellations. Conversely, among participants with counter-stereotypical associations (*d* < 0), such a scenario would elicit lower expectations.

Model (iv) examines whether the effect of disconfirmation on evaluations depends on congruity and whether this relationship is further moderated by participants’ gender-domain associations. In line with our main analyses (Section 5.2), we find no significant interaction between positive disconfirmation (i.e., underestimation) and congruity (β = −.096, *p* = .631), nor does this relationship depend on the *d*-score (β = .547, *p* = .211). These findings further speak against H2a.

**Fig 9:**
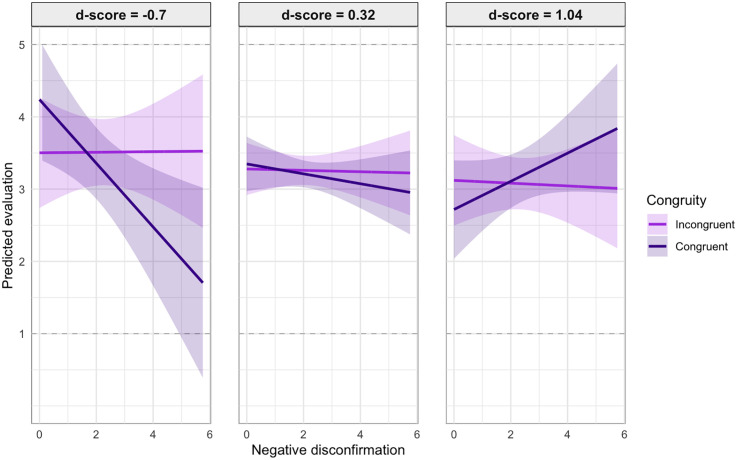
Effect of negative disconfirmation by congruity and d-score (with 95% confidence intervals), based on Model (iv).

For negative disconfirmation (i.e., overestimation), however, the interaction with congruity is significant (β = −.179, *p* = .035), suggesting that failing to meet expectations is penalized more harshly when workers operate in stereotypically congruent domains. Yet, accounting for the significant three-way interaction between negative disconfirmation, congruity, and *d*-score (β = .377, *p* = .036), the harsher penalty for underperformance in congruent domains is mitigated when participants have stronger stereotypical associations (reflected by positive *d*-scores). Fig 9 illustrates the relationship between negative disconfirmation and congruity at the minimum (*d* = −0.7), average (*d* = 0.32), and the maximum (*d* = 1.04) *d*-score values observed in our dataset.

In other words, participants whose associations follow stereotypical gender-domain patterns more strongly appear less strict when a congruent worker clearly underperforms (see right plot in Fig 9). In contrast, participants with counter-stereotypical associations tend to penalize such workers more severely (see left plot in Fig 9). These findings offer partial support for H2b. Whether the effect of disconfirmation on evaluations depends on congruity further hinges on the strength and direction of the individual’s associations. Only for sufficiently strong stereotypical associations, workers in congruent domains failing to meet expectations are rated more leniently than those perceived as incongruent. This would be consistent with H2b.

### 5.5. Subsample analysis

Based on these results, we considered whether the hypothesized positive effect of congruity on expectations only emerges for participants with stereotypical gender-domain associations. To explore this, we conducted a subsample analysis by dividing participants based on their IAT results into those with negative *d*-scores (indicating counter-stereotypical gender-domain associations) and those with positive scores (indicating stereotypical associations). Model (v) in Table 7 shows that for the small subgroup with negative *d*-scores, congruity had a negative effect on expectations (β = .717, *p* = .015).

**Table 7:** OLS regression analysis with expectation as dependent variable, for subsamples based on *d*-score and age.

	(v)	(vi)	(vii)	(viii)
	*d* < 0	*d* ≥ 0	> 25 years	≤ 25 years
Congruity	−.717* (.273)	.255* (.115)	.019 (.147)	.364* (.163)
Worker female	.623* (.265)	.117 (.115)	.007 (.146)	.386* (.162)
Participant female	−.444 (.265)	−.443*** (.115)	−.401** (.145)	−.405* (.159)
Gender match ^1)^	−.511 (.271)	.057 (.115)	.202 (.147)	−.217 (.157)
Constant	6.053*** (.307)	5.731*** (.124)	5.795*** (.148)	5.646*** (.186)
**Observations** ^2)^	28	167	93	102
**Adjusted *R*** ^**2**^	.300	.093	.052	.136
**Res. std. error**	.700 (*df *= 23)	.738 (*df *= 162)	.694 (*df *= 88)	.788 (*df* = 97)
***F*-statistic**	3.895 * (*df* = 4; 23)	5.274 *** (*df *= 4; 162)	2.265 (*df* = 4; 88)	4.961 ** (*df* = 4; 97)

**Note:** Standard errors in parentheses; + *p* < .1; *p < .05; **p < .01; ***p < .001

1)Binary variable indicating whether participant and worker were of the same gender.

2)For three observations, information on gender was not available.

Such a finding is plausible, given that these participants likely perceive stereotypical pairings (i.e., male-cars, female-cars) as incongruent, leading to lower expectations. Conversely, Model (vi) reveals that for participants with positive *d*-scores, stereotypical congruity had a positive effect on expectations, in line with H1 (β = .255, *p* = .027). These findings highlight that individual variations in gender-domain associations may account for the inconsistent or null effects of congruity observed in the overall sample.

Next, we considered whether the relatively young age of our sample might also help explain the null effects observed in our main analysis. To explore this, we conducted a subsample analysis using a median split at age 25. The findings from Models (vii) and (viii) in 7 suggest that among participants older than 25 years, congruity had no significant effect on expectations. In contrast, younger participants reported significantly higher expectations for workers in congruent domains (β = 0.364, *p* = .028). Additionally, within the younger subsample and the subgroup with counter-stereotypical gender-domain associations, participants held higher expectations for female workers. Models (v)-(viii) also indicate that female participants seem to have lower expectations, which does not systematically differ by strength of stereotypical associations (i.e., *d*-score) or age groups.

In addition, we explored whether the hypothesized interaction between disconfirmation and congruity only affected evaluations in certain subgroups. Once again, we used subsamples based on *d*-score and participant age. However, the hypotheses were not supported in either subgroup (see supporting information, S6 Table).

### 5.6. Post-hoc power analysis

Given the (partial) lack of support for our hypotheses, we conducted a post-hoc power analysis to assess the sensitivity of our study design. For H1, we estimated the effect size of congruity on expectations based on the observed differences in group means, yielding a small effect of *Cohen’s d* = .197. Using G*Power [62], we calculated the achieved power for a two-tailed independent samples *t*-test with our actual sample size (*n* = 198). The analysis revealed an achieved power of .28 which is significantly below the conventional threshold of .80. To detect an effect of this magnitude (*d* = .197) with 80% power at a significance level of α = .05, a total sample of *N* = 812 participants would have been required.

For H2a and H2b, which concerned the interaction effects between congruity and disconfirmation, we used a regression-based power analysis. Although G*Power does not offer a direct test for individual coefficients in multiple regression, it allows approximation via an *F*-test in *R*^*2*^ change. This method evaluates whether the inclusion of an interaction term significantly improves model fit beyond the main effects. Based on our data, the effect sizes were estimated at *f*^*2*^ = .001 (H2a) and *f*^*2*^ = .002 (H2b). The corresponding achieved powers were .07 and .10, respectively. Detecting effects of this magnitude with 80% power at α = .05 would have required sample sizes of *N* = 7,842 and 3,927 for H2a and H2b, respectively.

## 6. Discussion

Our study yields several insights into how expectations are formed and how their disconfirmation shapes subsequent evaluations. First, we find that expectations about workers do not significantly differ based on stereotypical worker-domain congruity (H1), contrary to what might be expected based on RCT. Moreover, expectations themselves do not seem to influence evaluations directly. Interestingly, performance exceeding expectations can have a negative effect on evaluations, suggesting an anchoring effect of relatively low expectations. These findings challenge some of the key tenets of EDT. Additionally, we find no evidence that these disconfirmation effects are affected by congruity (H2). Specifically, positive disconfirmation (i.e., underestimation of performance) is not rewarded more strongly for workers in congruent constellations (H2a), nor is negative disconfirmation (i.e., overestimation of performance) penalized more harshly for workers in stereotypically incongruent domains (H2b). In this section, we discuss our results in light of prior literature and highlight theoretical and practical implications. Lastly, we acknowledge the limitations of our study and propose directions for future research.

### 6.1. Theoretical implications

RCT predicts that incongruity between domain and gender will lead to lower expectations. Our study challenges and refines this perspective by identifying individuals’ implicit gender-domain associations and age as relevant boundary conditions.

First, we show that implicit gender-domain associations significantly condition congruity-based bias. Individuals with strong stereotypical associations hold biased expectations favoring workers in congruent domains and rate their underperformance more leniently than that of workers in congruent domains. Conversely, participants with counter-stereotypical gender-domain associations anticipate higher performance from workers in stereotypically incongruent domains and seem to apply milder standards when expectations are not met, compared to when workers in stereotypically congruent domains fail to meet expectations. In contrast, those with weak gender-domain associations do not exhibit congruity bias. By identifying implicit cognitive schemas as predictors of such bias, we introduce another layer of nuance into RCT, complementing previously recognized moderators such as individuals’ gender and cultural context.

Moreover, we identify age as another important boundary condition. Participants below 25 years exhibit stronger congruity-based bias than older individuals, a finding initially counterintuitive given broader societal trends towards gender egalitarianism and evidence suggesting younger individuals often endorse less rigid stereotypes [63]. Nevertheless, younger participants may also carry implicit bias acquired from early-life socialization, media exposure, and observation of labor-market inequalities [9]. This result reveals that implicit bias can subtly shape expectations even among groups explicitly endorsing egalitarian ideals.

In sum, we extend RCT by revealing boundary conditions under which congruity-based effects vary or even reverse. Future research should explicitly account for participants’ implicit gender-domain associations and age differences when investigating stereotype effects, rather than assuming homogenous patterns of bias.

Second, our study contributes to reconciling previously mixed findings on gender bias in the platform economy by integrating RCT and EDT. Prior studies show inconsistent evidence, with some reporting clear gender disparities in evaluations and hiring, while others report insignificant or opposite effects. We propose that these inconsistencies may arise because earlier studies have not considered the interplay between expectations, disconfirmation, and implicit gender-domain associations. Specifically, our findings indicate that congruity-based bias can already manifest in expectations, even before observable interactions on platforms occur. Such biased expectations, particularly among individuals with strong stereotypical gender-domain associations, can lead directly to biased hiring decisions, offering a coherent explanation for the results documented by Galperin [25], Chan and Wang [24], and Leung and Koppmann [26].

Furthermore, our findings shed light on why congruity-based bias predominantly emerges in contexts of low performance rather than high performance [6,7]. Specifically, we show that – given stereotypical gender-domain associations – individuals disproportionately penalize workers in incongruent constellations when their performance negatively disconfirms expectations. However, exceeding expectations does not lead to correspondingly stronger rewards for congruent workers. This asymmetry may explain why biased evaluations mainly occur in response to low performance (i.e., where negative disconfirmation is more likely). Our results also help reconcile the null effects reported by Thebault-Spieker et al. [35]. In their experiment, participants were asked to rate a worker who had been hired to critique a high school student’s essay. This task could be perceived as relatively gender neutral, limiting the potential for congruity-based bias. Similarly, the inconsistent findings reported by Hannák et al. [5] across different job domains may be explained by variation in the salience of gender stereotypes across those domains. Heterogeneity in participants’ implicit gender-domain associations could dampen aggregate-level effects and contribute to mixed results.

### 6.2. Practical implications

Our research has practical implications for various stakeholders in OLMs, including workers, clients, and platform providers. First, workers should aim for high performance while managing expectations, particularly those in incongruent domains, who can face lower expectations if clients hold stereotypical gender-domain associations. Workers should avoid being overly modest and engage in moderate self-promotion to raise expectations and minimize disconfirmation.

Second, clients should create conditions that enable workers to avoid disconfirmation, for instance, by communicating expectations clearly and early on. Setting clear, achievable goals and providing detailed job descriptions can help workers understand what is expected of them. Furthermore, clients should provide constructive feedback regularly along the way to help workers understand potential deviations from said expectations and thus enable them to more effectively work towards meeting them.

Third, our findings hold implications for platform providers who play a critical role in shaping the environment in which workers are evaluated. To reduce the salience of factors such as gender, platforms could, for instance, allow workers to use pseudonyms instead of first names. Regarding the use of photos, platforms face a trade-off: While profile photos can promote trust, they also enable the identification of gender and can serve as an anchor for stereotypical evaluation. To address this, platforms could encourage the use of logos or other non-personal images instead of profile photos, which can help maintain professionalism and reduce bias based on appearance.

### 6.3. Limitations and future research

This study has several limitations that should be acknowledged but they also open up avenues for future research. First, as with all scenario-based experiments, concerns about external validity remain. Participants were presented a stylized scenario that does not fully capture the complexity of real-world evaluation contexts, and their responses may therefore not fully reflect the behavior of clients operating in online labor markets. While participants, on average, rated the scenario as fairly realistic, self-reports in a vignette-based task may differ from actual decision-making in high-stakes, real-life settings. Future research could better approximate real client behavior by replicating this study in field settings or by employing experimental designs that introduce higher personal or financial stakes.

Second, our manipulation of congruity may not have been strong enough for the particular scenario and task – at least in relation to the general level of noise in the main variables. Future work should thus aim to identify even stronger triggers for (in-) congruity, for instance, by piloting different scenarios on their respective target group.

Third, participants were instructed to assume the role of an online shop manager, a framing that may constrain the generalizability of the findings to other types of platform users, particularly private individuals or clients from different industry contexts. Future research could also consider how scenario framing in experiments, but also individual attributes and situational contexts, shape expectations and their subsequent effect on evaluation.

Fourth, our measurement of participants’ gender-domain associations using the IAT comes with methodological challenges. While our exploratory analyses suggest some meaningful patterns, the IAT has been criticized for its low test-retest reliability at the individual level, which could undermine the stability of our categorization of participants into subgroups with stereotypical versus counter-stereotypical gender-domain associations. Therefore, our findings should be interpreted with caution. Future research should explore alternative or complementary methods of assessing gender-domain associations to replicate and extend our findings.

Fifth, we did not measure disconfirmation directly but rather inferred it by calculating the difference between participants’ stated expectations and the worker’s assigned performance. However, this operationalization assumes that individuals perceive disconfirmation in a linear and symmetrical manner. As such, our inferred disconfirmation may over- or underestimate participants’ *perceived* disconfirmation. Future research should therefore measure both expectations and disconfirmation as separate constructs.

Lastly, the statistical power of our study was limited. Post-hoc power analyses indicate that our sample size may have been insufficient to detect the hypothesized effects, especially in models including interaction terms. As such, our results remain somewhat inconclusive, as it is possible that the predicted effects do exist but were too small to be detected with our selected sample size. Larger-scale replications are necessary to clarify whether the lack of significant effects reflects a true null finding or results from power limitations in this study.

## 7. Conclusion

An excellent reputation is crucial for workers in OLMs as it directly affects demand for their services and their wages. Therefore, ensuring fair evaluations of workers, irrespective of their gender and stereotypical role ascriptions, is essential for establishing equal opportunities.

Our findings reveal no systematic congruity-based bias at the aggregate level but conditional effects depending on the strength and direction of gender-domain associations. Given stereotypical associations, perceived congruity between workers’ gender and domain increases clients’ expectations about workers’ performance. When workers fall short of these expectations, those perceived as congruent are evaluated more leniently than those in incongruent settings. This suggests that individuals with strong gender-domain associations are particularly susceptible to congruity-based bias when their expectations are not met.

Thus, our study contributes to a more nuanced understanding of how congruity-based disparities can emerge on online platforms, highlighting the critical role of managing expectations to create more equitable markets. By shedding light on the interplay between gender, domain, expectations, and disconfirmation, our study paves the way for developing strategies to make evaluations more objective and fairer overall.

## Supporting information

S1 Fig Stimulus material, profile pictures for female workers (AI-generated). (TIF)

S2 Fig Stimulus material, profile pictures for male workers (AI-generated). (TIF)

S1 Table Perceived characteristics based on profile pictures. (DOCX)

S2 Table Performance descriptions of worker in email. (DOCX)

S3 Table Descriptive statistics. *Note:* Mean values, standard deviation in parentheses; ^1)^ Expectation: 1 = min, 7 = max; ^2)^ Rating: 1.0 = min, 5.0 = max.
(DOCX)

S4 Table OLS regression results with expectation as the dependent variable, including gender, domain, and their interaction. *Note:* Standard errors in parentheses; ^+^ *p *< .10; *p < .05; **p < .01; ***p < .001; ^1)^ Ethnicity “White” as baseline; ^2)^ Binary variable indicating whether participant and worker were of the same gender; ^3)^ For three observations, information on gender was not available.
(DOCX)

S5 Table OLS regression results with expectation as the dependent variable, including gender, domain, and their interaction. *Note:* Standard errors in parentheses; ^+^ *p *< .10; *p < .05; **p < .01; ***p < .001; ^1)^ Ethnicity “White” as baseline; ^2)^ Binary variable indicating whether participant and worker were of the same gender; ^3)^ For three observations, information on gender was not available.
(DOCX)

S6 Table OLS regression results with evaluation as the dependent variable, for subsamples based on *d*-score and age. *Note:* Standard errors in parentheses; ^+^ *p *< .10; *p < .05; **p < .01; ***p < .001; ^1)^ Ethnicity “White” as baseline; ^2)^ Binary variable indicating whether participant and worker were of the same gender; ^3)^ For three observations, information on gender was not available.
(DOCX)
