# Supplementary figures and images for: Expectation vs. reality: How stereotypes and expectation disconfirmation affect job evaluations in online labor markets
Source: PLoS One. 2025 Nov 4;20(11):e0334630. doi: 10.1371/journal.pone.0334630 (PMC12585043; doi:10.1371/journal.pone.0334630)

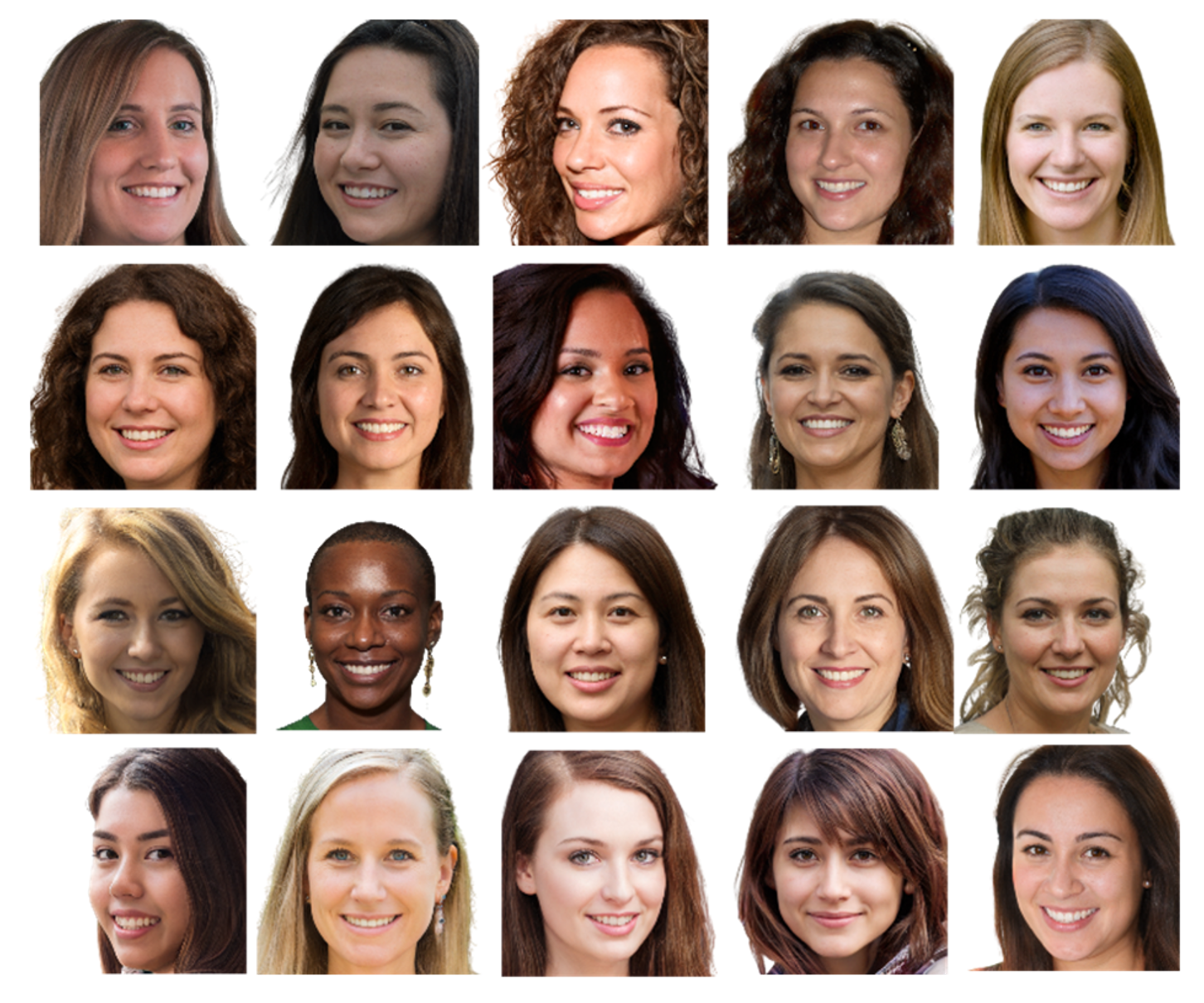

Supplement: S1 Fig — (TIF) [file pone.0334630.s001.tif]

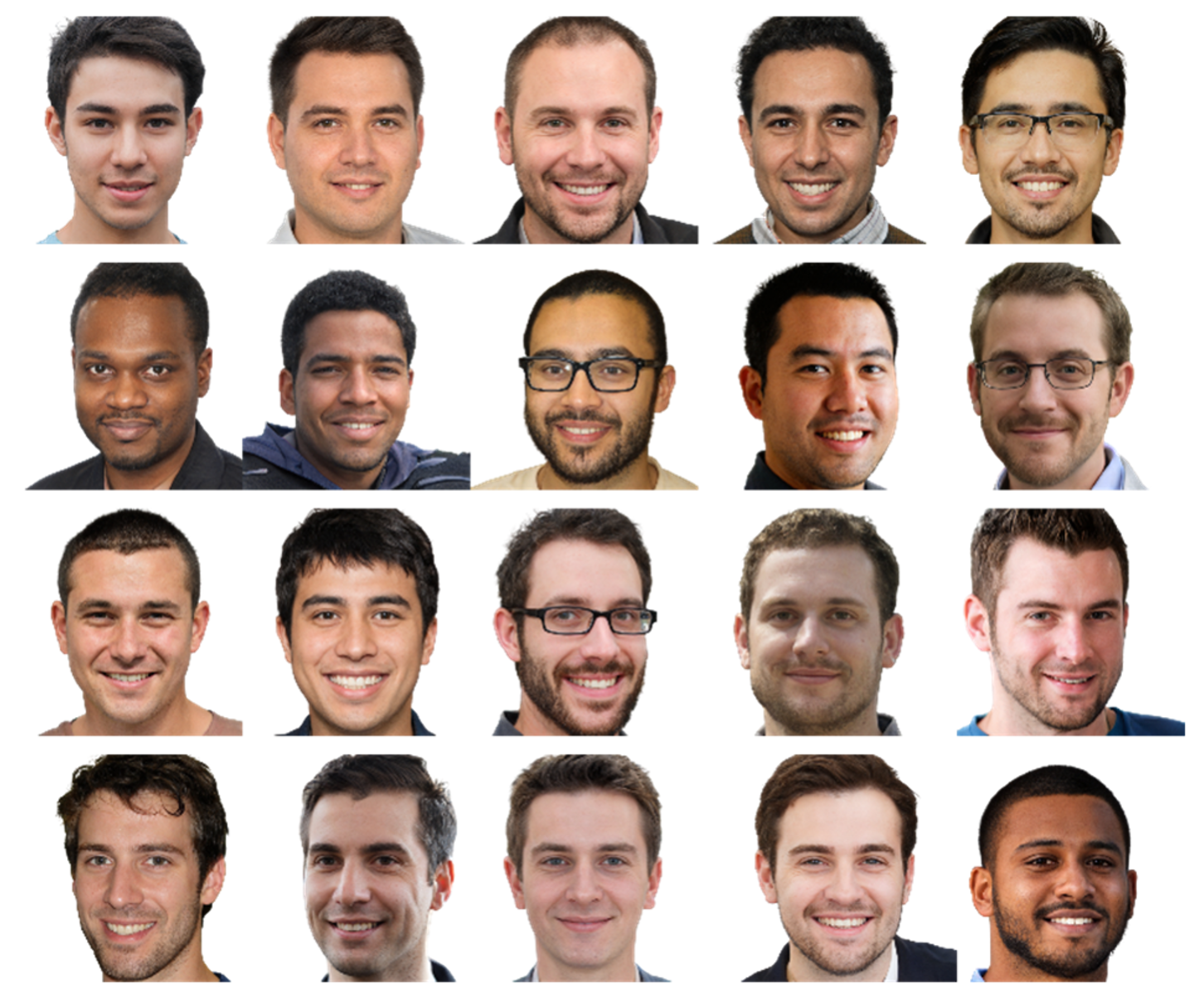

Supplement: S2 Fig — (TIF) [file pone.0334630.s002.tif]
