# Supplementary material for: Expectation vs. reality: How stereotypes and expectation disconfirmation affect job evaluations in online labor markets
Source: PLoS One. 2025 Nov 4;20(11):e0334630. doi: 10.1371/journal.pone.0334630 (PMC12585043; doi:10.1371/journal.pone.0334630)
Supplement: S1 Table — (DOCX) [file pone.0334630.s003.docx]

| **S1 Table**: Perceived characteristics based on profile pictures | | | | | | | | |
| --- | --- | --- | --- | --- | --- | --- | --- | --- |
| Worker | Domain | Age | Attrac-tive-ness | Compe-tence | Happi-ness | Trust-worthi-ness | Warmth | Race |
| Male | Car | 30.40 (3.02) | 4.28 (0.36) | 4.95 (0.31) | 5.01 (0.42) | 4.68 (0.37) | 4.68 (0.34) | 74.55% White  3.64% Asian  14.55% Black  7.27% Hispanic |
|  | Fashion | 28.65 (3.76) | 4.60 (0.54) | 4.81 (0.31) | 5.24 (0.40) | 4.77 (0.39) | 5.04 (0.50) | 63.27% White  16.33% Asian  8.16% Black  12.24% Hispanic |
| Female | Car | 29.91 (4.13) | 4.39 (0.36) | 4.87 (0.31) | 5.03 (0.42) | 4.60 (0.40) | 4.66 (0.40) | 68.63% White  1.96% Asian  11.76% Black  17.65% Hispanic |
|  | Fashion | 30.51 (3.51) | 4.56 (0.42) | 4.84 (0.44) | 5.17 (0.35) | 4.70 (0.41) | 5.05 (0.48) | 72.09% White  9.30% Asian  2.33% Black  16.28% Hispanic |
