# Supplementary material for: Expectation vs. reality: How stereotypes and expectation disconfirmation affect job evaluations in online labor markets
Source: PLoS One. 2025 Nov 4;20(11):e0334630. doi: 10.1371/journal.pone.0334630 (PMC12585043; doi:10.1371/journal.pone.0334630)
Supplement: S2 Table — (DOCX) [file pone.0334630.s004.docx]

| **S2 Table**: Performance descriptions of worker in email | | |
| --- | --- | --- |
| Punctuality | Low | After $name hadn't submitted $his/her work on Wednesday as agreed upon, we reached out to $him/her. $he/she_cap made some excuses but eventually handed in the work (although a week later than hoped). I was quite mad because $he/she didn't even give us a notice although I repeatedly told $him/her that we need the results by Wednesday. |
|  | Medium | $name has submitted $his/her work a few days late although I had repeatedly told $him/her that we need the results by Wednesday. Well, at least $he/she had given us a notice a week in advance that this might happen. |
|  | High | I was a bit nervous whether $name could manage to deliver on time but $he/she did submit $his/her work punctually on Wednesday. |
| Layout | Low | Overall, I must say that, unfortunately, the website's layout and aesthetics do not comply with the guidelines we had specified. $name's design was rather ordinary and mediocre compared to those of our competitors. I was disappointed about $his/her ideas. |
|  | Medium | Overall, the website's layout and aesthetics mostly comply with the guidelines we had specified. $name's design was good, but the designs are actually quite similar to those of our competitors. All in all, I was satisfied with $his/her ideas. |
|  | High | Overall, the website's layout and aesthetics fully comply with the guidelines we had specified. $name's design was very creative and actually superior to those of our competitors. I was quite thrilled about $his/her ideas! |
| Product descriptions | Low | The product descriptions were often imprecise and had several obvious, careless mistakes. I wonder how this could happen in times of automatic spell checkers. Although $name's text reflected $his/her knowledge about $domain, $his/her writing did not get me too excited about the products. |
|  | Medium | The product descriptions were well-written and mostly error-free (with a few issues for us left to fix). $name's text reflected $his/her good knowledge about $domain. Overall, $name's texts were informative and adequate for our target customer but sometimes too colloquial. |
|  | High | The product descriptions were eloquent and error-free (with only a few minor issues for us left to fix). $name's text reflected $his/her expertise in $domain. $his/her_cap texts were convincing, enthusiastic, and perfectly addressed our target customer. |
| Conclusion | Low | To sum up, we still need to fix a considerable number of major mistakes and redo many design features. I guess we'll need a week, so we'll be quite off our time target… |
|  | Medium | To sum up, we need to fix a few minor mistakes and improve some design features. I guess we'll need three days, so we'll miss our time target by a bit… |
|  | High | To sum up, there is only little potential for improvement. I guess we'll need a day for some last changes, so we'll miss our time target by a bit. |
