# Supplementary material for: Expectation vs. reality: How stereotypes and expectation disconfirmation affect job evaluations in online labor markets
Source: PLoS One. 2025 Nov 4;20(11):e0334630. doi: 10.1371/journal.pone.0334630 (PMC12585043; doi:10.1371/journal.pone.0334630)
Supplement: S3 Table — Note: Mean values, standard deviation in parentheses; 1) Expectation: 1 = min, 7 = max; 2) Rating: 1.0 = min, 5.0 = max. (DOCX) [file pone.0334630.s005.docx]

| **S3 Table**: Descriptive statistics | | | | | |
| --- | --- | --- | --- | --- | --- |
| Worker | Domain | Congruity | # of observations | Expectation ^1)^ | Rating ^2)^ |
| Male | Car | Yes | 55 | 5.74 (0.82) | 3.29 (1.26) |
|  | Fashion | No | 51 | 5.51 (0.86) | 3.02 (1.15) |
| Female | Car | No | 49 | 5.75 (0.67) | 3.18 (1.12) |
|  | Fashion | Yes | 43 | 5.84 (0.72) | 3.41 (1.19) |
| **Note:** Mean values, standard deviation in parentheses  ^1)^ Expectation: 1 = min, 7 = max  ^2)^ Rating: 1.0 = min, 5.0 = max | | | | | |
