# Supplementary material for: Expectation vs. reality: How stereotypes and expectation disconfirmation affect job evaluations in online labor markets
Source: PLoS One. 2025 Nov 4;20(11):e0334630. doi: 10.1371/journal.pone.0334630 (PMC12585043; doi:10.1371/journal.pone.0334630)
Supplement: S4 Table — Note: Standard errors in parentheses; + p < .10; *p < .05; **p < .01; ***p < .001; 1) Ethnicity “White” as baseline; 2) Binary variable indicating whether participant and worker were of the same gender; 3) For three observations, information on gender was not available. (DOCX) [file pone.0334630.s006.docx]

| **S4 Table**: OLS regression results with expectation as the dependent variable, including gender, domain, and their interaction | | | | | | | | |
| --- | --- | --- | --- | --- | --- | --- | --- | --- |
|  | M1 | | M2 | | M3 | | M4 | |
| Worker female | 0.157  (0.111) |  | 0.213  (0.118) | ^+^ | 0.009  (0.152) |  | 0.130  (0.156) |  |
| Domain fashion | -0.076  (0.111) |  | -0.081  (0.109) |  | -0.222  (0.151) |  | -0.158  (0.144) |  |
| Worker female × Domain fashion |  |  |  |  | 0.314  (0.221) |  | 0.176  (0.214) |  |
| Worker age |  |  | -0.012  (0.018) |  |  |  | -0.014  (0.018) |  |
| Worker attractiveness |  |  | -0.207  (0.150) |  |  |  | -0.202  (0.150) |  |
| Worker competence |  |  | 0.070  (0.190) |  |  |  | 0.069  (0.190) |  |
| Worker happiness |  |  | 0.286  (0.157) | ^+^ |  |  | 0.295  (0.158) | ^+^ |
| Worker trustworthiness |  |  | 0.284  (0.162) | ^+^ |  |  | 0.283  (0.163) | ^+^ |
| Worker warmth |  |  | -0.198  (0.150) |  |  |  | -0.207  (0.151) |  |
| Worker ethnicity ^1) Asian^ |  |  | -0.028  (0.223) |  |  |  | -0.020  (0.223) |  |
| ^Black^ |  |  | -0.105  (0.205) |  |  |  | -0.093  (0.206) |  |
| ^Hispanic^ |  |  | -0.133  (0.166) |  |  |  | -0.126  (0.166) |  |
| Participant age |  |  | 0.002  (0.007) |  |  |  | 0.002  (0.007) |  |
| Participant ethnicity ^1) Asian^ |  |  | -0.677  (0.337) | * |  |  | -0.656  (0.338) |  |
| ^Black^ |  |  | 0.548  (0.146) | *** |  |  | 0.532  (0.147) | *** |
| ^Mixed^ |  |  | 0.210  (0.197) |  |  |  | 0.202  (0.198) |  |
| ^Other^ |  |  | 0.118  (0.311) |  |  |  | 0.111  (0.311) |  |
| Participant female |  |  | -0.303  (0.111) | ** |  |  | -0.302  (0.111) | ** |
| Gender match ^2)^ |  |  | 0.045  (0.106) |  |  |  | 0.047  (0.106) |  |
| Constant | 5.666  (0.092) | *** | 4.756  (1.710) | ** | 5.736  (0.105) | *** | 4.832  (1.714) | ** |
| **Observations** | 198 | | 195 ^3)^ | | 198 | | 195 ^3)^ | |
| **Adjusted *R*^2^** | 0.003 | | 0.164 | | 0.008 | | 0.162 | |
| **Res. std. error** | 0.778  (df = 195) | | 0.717  (df = 176) | | 0.776  (df = 194) | | 0.718  (df = 175) | |
| ***F*-Statistic** | 1.256  (df = 2; 195) | | 3.113***  (df = 18; 176) | | 1.512  (df = 3; 194) | | 2.979***  (df = 19; 175) | |
| **Note:** Standard errors in parentheses; ^+^ *p* < .10; **p* < .05; ***p* < .01; ****p* < .001  ^1)^ Ethnicity “White” as baseline  ^2)^ Binary variable indicating whether participant and worker were of the same gender  ^3)^ For three observations, information on gender was not available. | | | | | | | | |
