# Supplementary material for: Expectation vs. reality: How stereotypes and expectation disconfirmation affect job evaluations in online labor markets
Source: PLoS One. 2025 Nov 4;20(11):e0334630. doi: 10.1371/journal.pone.0334630 (PMC12585043; doi:10.1371/journal.pone.0334630)
Supplement: S5 Table — Note: Standard errors in parentheses; + p < .10; *p < .05; **p < .01; ***p < .001; 1) Ethnicity “White” as baseline; 2) Binary variable indicating whether participant and worker were of the same gender; 3) For three observations, information on gender was not available. (DOCX) [file pone.0334630.s007.docx]

| **S5 Table**: OLS regression results with rating as the dependent variable, including gender, domain, and their interaction | | | | | | | | |
| --- | --- | --- | --- | --- | --- | --- | --- | --- |
|  | M5 | | M6 | | M7 | | M8 | |
| Worker female | 0.127  (0.169) |  | 0.231  (0.199) |  | -0.117  (0.233) |  | 0.034  (0.261) |  |
| Domain fashion | -0.038  (0.169) |  | -0.108  (0.182) |  | -0.277  (0.230) |  | -0.292  (0.241) |  |
| Worker female × Domain fashion |  |  |  |  | 0.515  (0.338) |  | 0.419  (0.359) |  |
| Worker age |  |  | 0.043  (0.031) |  |  |  | 0.040  (0.031) |  |
| Worker attractiveness |  |  | -0.078  (0.251) |  |  |  | -0.066  (0.251) |  |
| Worker competence |  |  | 0.240  (0.319) |  |  |  | 0.238  (0.319) |  |
| Worker happiness |  |  | 0.161  (0.264) |  |  |  | 0.182  (0.264) |  |
| Worker trustworthiness |  |  | -0.444  (0.272) |  |  |  | -0.447  (0.272) |  |
| Worker warmth |  |  | -0.180  (0.252) |  |  |  | -0.201  (0.252) |  |
| Worker ethnicity ^1) Asian^ |  |  | 0.351  (0.374) |  |  |  | 0.370  (0.374) |  |
| ^Black^ |  |  | -0.056  (0.344) |  |  |  | -0.027  (0.345) |  |
| ^Hispanic^ |  |  | -0.055  (0.279) |  |  |  | -0.039  (0.279) |  |
| Participant age |  |  | 0.006  (0.012) |  |  |  | 0.005  (0.012) |  |
| Participant ethnicity ^1) Asian^ |  |  | -0.406  (0.565) |  |  |  | -0.358 (0.566) |  |
| ^Black^ |  |  | 0.126  (0.244) |  |  |  | 0.088  (0.246) |  |
| ^Mixed^ |  |  | 0.288  (0.331) |  |  |  | 0.269  (0.331) |  |
| ^Other^ |  |  | -0.235  (0.521) |  |  |  | -0.254  (0.521) |  |
| Participant female |  |  | 0.145  (0.187) |  |  |  | 0.148  (0.187) |  |
| Gender match ^2)^ |  |  | 0.141  (0.178) |  |  |  | 0.144  (0.177) |  |
| Constant | 3.178  (0.141) | *** | 2.856  (2.870) |  | 3.293  (0.160) | *** | 3.035  (2.871) |  |
| **Observations** | 198 | | 195 ^3)^ | | 198 | | 195 ^3)^ | |
| **Adjusted *R*^2^** | -0.007 | | -0.019 | | -0.0003 | | -0.017 | |
| **Res. std. error** | 1.189  (df = 195) | | 1.203  (df = 176) | | 1.185  (df = 194) | | 1.202  (df = 175) | |
| ***F*-Statistic** | 0.309  (df = 2; 195) | | 0.801  (df = 18; 176) | | 0.982  (df = 3; 194) | | 0.832  (df = 19; 175) | |
| **Note:** Standard errors in parentheses; ^+^ *p* < .10; **p* < .05; ***p* < .01; ****p* < .001  ^1)^ Ethnicity “White” as baseline  ^2)^ Binary variable indicating whether participant and worker were of the same gender  ^3)^ For three observations, information on gender was not available. | | | | | | | | |
