# Supplementary material for: Expectation vs. reality: How stereotypes and expectation disconfirmation affect job evaluations in online labor markets
Source: PLoS One. 2025 Nov 4;20(11):e0334630. doi: 10.1371/journal.pone.0334630 (PMC12585043; doi:10.1371/journal.pone.0334630)
Supplement: S6 Table — Note: Standard errors in parentheses; + p < .10; *p < .05; **p < .01; ***p < .001; 1) Ethnicity “White” as baseline; 2) Binary variable indicating whether participant and worker were of the same gender; 3) For three observations, information on gender was not available. (DOCX) [file pone.0334630.s008.docx]

| **S6 Table**: OLS regression analysis with evaluation as dependent variable, for subsamples based on *d*-score and age | | | | | | | | |
| --- | --- | --- | --- | --- | --- | --- | --- | --- |
|  | M9 | | M10 | | M11 | | M12 | |
|  | *d* < 0 | | *d* ≥ 0 | | > 25 years | | ≤ 25 years | |
| Congruity | .872  (.544) |  | -.051  (.179) |  | .172  (.267) |  | -.050  (.210) |  |
| Performance | .728  (.270) | * | .513  (.074) | *** | .801  (.112) | *** | .362  (.086) | *** |
| Positive disconfirmation ^1)^ | -.365  (.248) |  | -.394  (.153) | * | -.404  (.202) | * | -.280  (.145) |  |
| Negative disconfirmation ^2)^ | .229  (.269) |  | -.059  (.081) |  | .291  (.114) | * | -.264  (.102) | * |
| Positive disconfirmation ^1)^  × Congruity | -.388  (.437) |  | .155  (.187) |  | .141  (.272) |  | .018  (.187) |  |
| Negative disconfirmation ^2)^  × Congruity | -.401  (.201) |  | .005  (.059) |  | -.072  (.087) |  | .025  (.074) |  |
| Worker is female | .204  (.358) |  | -.024  (.094) |  | -.017  (.132) |  | .018  (.122) |  |
| Participant female | .077  (.313) |  | .166  (.097) |  | .178  (.133) |  | .125  (.120) |  |
| Gender match ^3)^ | .188  (.291) |  | .022  (.093) |  | -.066  (.131) |  | .116  (.118) |  |
| Constant | -0.108  (1.666) |  | 1.298  (0.446) | ** | -0.556  (0.660) |  | 2.277  (0.521) | *** |
| **Observations** ^4)^ | 28 | | 167 | | 93 | | 102 | |
| **Adjusted *R*^2^** | .677 | | .753 | | .737 | | .775 | |
| **Res. std. error** | .675 (df = 18) | | .589 (df = 157) | | .603 (df = 83) | | .574 (df = 92) | |
| ***F*-statistic** | 7.287 ***  (df = 9; 18) | | 57.334 ***  (df = 9; 157) | | 29.655 ***  (df = 9; 83) | | 39.691 ***  (df = 9; 92) | |
| **Note:** Standard errors in parentheses; ^+^ *p* < .1; **p* < .05; ***p* < .01; ****p* < .001  ^1)^ Underestimation of performance  ^2)^ Overestimation of performance  ^3)^ Binary variable indicating whether participant and worker were of the same gender.  ^4)^ For three observations, information on gender was not available. | | | | | | | | |
